# Aural, Nasal and Laryngeal Tuberculosis—With Special Reference to the Adirondacks as a Winter Health Resort1Read at the twenty-eighth annual meeting of the Central New York Medical Association, held at Syracuse, October 15, 1895.

**Published:** 1895-12

**Authors:** Sargent F. Snow

**Affiliations:** Aurist and laryngologist to the House of the Good Shepherd, Shelter for Unprotected Girls and the Syracuse Free Dispensary; member of the Central New York Medical Association, the Syracuse Academy of Medicine, the Onondaga County Medical Society, etc.


### AURAL, NASAL AND LARYNGEAL TUBERCULOSIS—WITH SPECIAL REFERENCE TO THE ADIRONDACKS AS A WINTER HEALTH RESORT.[1]

By SARGENT F. SNOW, M. D.,

Aurist and laryngologist to the House of the Good Shepherd, Shelter for Unprotected Girls and the Syracuse Free Dispensary; member of the Central New York Medical Association, the Syracuse Academy of Medicine, the Onondaga County Medical Society, etc.

TUBERCULOSIS of the ear, nose and throat is a subject too broad in its entirety to admit of an exhaustive paper that can be read in the time allotted to me. I trust, then, that you will pardon brevity in my treatment of some of the components of this article and my evident haste to get to the latter part of the subject, *i. e.*, laryngeal tuberculosis and the Adirondacks as a winter health resort.

Aural tuberculosis, the first on my list, is a comparatively rare disease, and upon which there appears to be a very small amount of literature. This latter fact may be accounted for if we remember that it usually comes in the course of general tuberculosis and is not thought worthy of much notice, except we be looking for every possible source of tubercular infection.

Hartman claims, in his hand-book, "That we may explain general tuberculosis originating in the ear from the entrance of tubercular bacilli from the outside into the deposit of pus, which find there a favorable soil, spread thence over the body and thus produce general infection." Direct transmission of tuberculosis, from the sputum to the mucous membrane of the tympanic cavity, may also take place through the Eustachian tube.

The symptoms of tubercular infection of the ear are commonly non-inflammatory; some deafness or discharge first attracts the patient's attention and then the aurist is consulted. Inspection, in

---

1. Read at the twenty-eighth annual meeting of the Central New York Medical Association, held at Syracuse, October 15, 1895.

the earlier stages of the trouble, will usually show more or less infiltration of the drum membrane and a distinctly circumscribed smooth nodule, of reddish appearance, about as large as a hemp seed. This first one will be followed by a second nodule, with the first one undergoing a disintegration, and later a complete perforation of the drum occurs. The treatment should be thorough cleansing and careful applications of a 40 to 80 per cent. lactic acid solution on a probe, and such constitutional remedies as are prescribed for the general tubercular condition that is usually present.

Tuberculosis in the nasal passages is more rare than in any other part of the respiratory tract—perhaps one in 300 cases. Bosworth states "That he finds but twenty-seven cases reported in literature." I, myself, have treated but one case, and that was of the hyperplastic form. The course of the disease is like the ordinary tubercular ulceration that is found in other portions of the air tract. Sometimes it shows itself in the form of small tumors, presenting a mammillated or raspberry appearance. The first described, or ulcerative form, usually comes on the septum or floor of the nares, while the hyperplastic form is more frequently found on the turbinated bodies. Tubercular ulceration, wherever it may be, presents certain distinguishing features. The surface is of a whitish-gray color and not depressed below the surrounding tissue. It is somewhat rounded, but irregular, while the mucous membrane bordering upon it appears normal. The secretion is usually of whitish-gray mucus, rarely ever pus, and, as a rule, more or less profuse. The hyperplastic form presents, on inspection, small projections from the membrane of the turbinated bodies of a reddish-gray tinge. They are usually much smaller, more flattened and more regularly rounded than a papillomatous growth. Fortunately, a tubercular process within the nose does not materially affect the prognosis of the general disease to which it is secondary.

As to treatment, little need be said. The ulcerated surface should be scraped and a lactid acid solution applied, or, in the mammillated variety, the snare may be used, with the idea of totally exterminating the disease, where it appears feasible.

Tubercular ulceration of the pharynx is also a rare disease, many authorities claiming it to be seen in only about 1 per cent. of tubercular patients, while tubercular manifestations in the larynx occur in from 25 to 30 per cent.

Both Fraenkel and Bosworth are inclined to the opinion that tuberculosis of the pharynx is a disease that may attack apparently healthy persons. There is a general view, however, that we may regard it as a manifestation of an *acute miliary* tuberculosis, the deposit occurring at the same time in the larynx, lungs and other parts of the body. In some instances, the disease appears late in the course of chronic tuberculosis. The appearance of a tubercular ulceration in the pharynx is characteristic, the ulcer being irregular in shape, very slightly raised, with poorly defined margins. Usually, in close proximity, can be seen small reddish-gray papulas, more or less confluent, and which will, within a few days, become an ulcerated area. In other cases, one or two isolated papulas may occur in an apparently healthy individual and remain latent for a long time. Tubercular ulceration of the pharynx and soft palate is a condition attended with many distressing symptoms, and, if not coincident with, is, certainly very soon after, followed by an invasion of the larynx. Along with the infiltration and ulceration there will be sharp, lancinating pains in the fauces, aggravated by deglutition and, in some cases, a rapid loss in the contractility of the palate and a consequent passage of food and drink into the nasal cavities.

Tubercular ulceration of this region may be mistaken for the superficial or deep ulceration of syphilis. Bosworth, in his recent work on the nose and throat, speaking of tuberculosis of the pharynx, says :

> The three appearances which I regard as important and somewhat pathognomic are the flush surface of the ulcer, the ropy mucous secretion and the almost uniform color of the ulcerated surface and its surrounding membrane. In syphilis, on the other hand, we have a rapid destruction of tissue in connection with active, localised inflammatory action. Hence, the secretion from the syphilitic ulcer is largely purulent ; it contains a certain amount of necrotic tissue, according to the rapidity of the ulcerative action. The ulcerated surface is notably hyperemic, while the mucous membrane surrounding it shows a well-marked areola, according to the extent of the gummatous infiltration from which it originally resulted.

Added to this, we have the fact that with the tubercular ulceration there are more or less systemic disturbances, which are absent in the later stages of syphilis.

The local treatment of these tubercular ulcerations consists in clearing off the surface with some alkaline spray, after which lactic acid should be thoroughly rubbed in. Some anodyne spray, as a 2 to 4 per cent. solution of cocaine, may be used, especially before eating, to relieve the pain of swallowing.

Tuberculosis of the larynx is a more common but less virulent disease than tuberculosis of the pharynx, and takes on more of the symptoms that characterise pulmonary phthisis, which it usually accompanies, than of acute miliary tuberculosis. As we have said before, the *larynx* is involved in from 25 to 30 per cent. of the cases of pulmonary tuberculosis.

Virchow recommends it as the most appropriate place for the study of tubercules. To distinguish laryngeal tuberculosis from other diseases in the same region, Lennox Browne, in a very trite way, says: "It is quite certain that the pale, opaque tumefaction of the arytenoid cartilages, and the epiglottis in laryngeal phthisis, has not the clear transparency of serous edema, the active glandular inflammation of simple laryngitis, the hyperplastic infiltration of syphilis or the angry inflammatory irritation of carcinoma."

We should add that in some patients with general tuberculosis, a laryngitis may occur, which is not tubercular and in no way dependent upon tubercules within the larynx. It differs from simple laryngitis by being more intractable and more apt to recur.

The symptoms and general appearance of tuberculosis of the larynx I will describe by a review of two of my cases, and mention the treatment I have found to give best results:

March 6, 1893, Miss M., aged 18, was referred to me by Dr. A. B. Miller, for hoarseness and a suspected development of phthisis. This young woman claimed that her general health was perfect up to ten months previous, when a slight cough began which had continued as slight until January, when she took a severe cold. This was followed by hoarseness, cessation of the menses and night sweats. An examination of the larynx showed the right vocal band comparatively clear and white, but the left cord was red, thickened and rough. The interarytenoid space was somewhat infiltrated, but there was no tumefaction of arytenoid cartilages or epiglottis.

Temperature, $100\frac{3}{5}°$; pulse, 112; respiration, 24. Physical examination of the chest disclosed nothing except a slight dulness and an increase in the expiratory sound, just below the right clavicle. I treated the patient with the usual constitutional remedies and the application of a 40 per cent. lactic acid solution to the ulcerated vocal band, thoroughly rubbed in three times a week, until April 8th, a period of one month. An examination then showed that the laryngeal trouble was almost removed, though a little thickening of the left cord and the tissues between the arytenoids remained. The voice was much improved, though not perfectly clear. The constitutional symptoms were, on the other hand, more aggravated. Temperature ranged a degree higher, sputum was rich with tubercular bacilli and a physical examination showed a marked increase in the dulness below the right clavicle, with the addition of mucus râles.

Upon consultation, it was decided that the patient should go to the Adirondacks, where she could have the advantages that a better altitude, dry atmosphere, porous soil and exercise in the open air would give. Specific directions as to diet, cold baths and the like were given, and April 21st the young woman started for the North Woods. I find by my record of her case that she returned here October 5th, after a five and a half months' absence. Her temperature ranged at 99°, pulse 90 and respiration 20; flesh hard, good color and feeling in perfect health. The vocal bands were about the same as at the time I last saw her, the ulceration being completely removed. Physical examination revealed the fact that the active process in the lungs was stopped and only a slight dulness could be found in the affected portion. One week later, she came in and her temperature registered a degree higher. There was some return of the cough and her appetite was beginning to fail. An immediate return to the woods was advised, but she could not be persuaded to go until the middle of November. During the five weeks' stay here at her home she run down rapidly, having lost six of the twelve pounds gained during the summer and a decidedly active tubercular process involving the upper lobe of the right lung was apparent.

At this time, November 14, 1893, through the kindness of Dr. Vadeboncouer, a patient came under my care, presenting many symptoms in common with the one just mentioned:

Miss W., aged 20, giving the same history as Case I., except that the voice was completely lost and the constitutional symptoms were more pronounced. Examination of the larynx showed that both vocal bands were deeply ulcerated. The tissue between the arytenoids was much infiltrated and their apices showed the pale bluish-red tumefaction peculiar to tuberculosis. Examination of the lungs revealed that there was marked consolidation of the left apex, with quite large and numerous mucus râles. The general appearance of the patient, as well as the history, and the like, told plainly that we had to deal with a case of unusual virulence, and that a change of climate was more imperative than local treatment. She was also advised to try the Adirondacks, though it was just in the beginning of winter. Arrangements were made as soon as possible, but it was not until December 6th that she took her departure. In the meantime, active measures were taken to stop the ulceration within the larynx, using a 60 per cent. solution of lactic acid, thoroughly rubbed in, with the result that the destructive process was stayed, though the infiltration between, and the swelling of, the arytenoids remained.

My next record of the two cases was made March 1st, when they informed me by a joint letter that the first, or Case I., weighed 128 pounds, a gain of thirteen pounds, and the second, or Case II., weighed 120 pounds, a gain of ten pounds in three and a half months.

May 1, 1894, I was trout fishing in that neighborhood, and I found that the first young lady was teaching school near by and was apparently well; but the one whom I sent last was losing ground. Upon looking into the matter, I learned that she, feeling as she expressed it, perfectly well, had neglected to take her cold baths and out-of-doors tramp each day, as had been directed. It was thought best to resume them and she soon began to regain the ground she had lost. When she returned to this city, August 1, 1894, I found her very much improved. Seemingly a good recovery, except the thickening of the vocal apparatus and some impaired breathing and dulness over the affected portion of the left lung. An examination, made October 15th, brought out the fact that the expected constitutional symptoms were returning, and, as circumstances did not seem to admit of a return to the woods, she soon began to fail and within three months died of pulmonary tuberculosis.

Case I., Miss M., taught school last winter, in the same region, and wrote me that she weighed 134 pounds, coughed but little and only when she took cold. Her voice was good but not perfectly clear. Called herself well. While on a short visit home, in February of this year, I examined her and found still a small consolidated area in the right lung, but no evidence of any active tubercular process. She is again teaching, and I venture to say that had she stayed in that climate continuously, and made no visits home, her recovery would have by this time been complete; though I should even now have to advise that she live in a climate suited to her until thirty-five or forty years of age.

I have selected notes from the two foregoing cases, merely because they represent so well the characteristic features of laryngeal tuberculosis and the many problems we meet in the treatment of such patients. As you remember, each one responded promptly to the local application to the larynx; but, in spite of our best efforts with these and the usual constitutional treatment, the pulmonary trouble increased until the patient was removed from the moisture-laden atmosphere of this section. As you noted also, each patient continued to improve while in a favorable climate taking her daily cold baths and out-of-door exercise; but even a visit home of one week would again start up the constitutional symptoms.

The best illustration of the comparative value of Adirondack air was shown when the two young women came home, August 1st, of last year, both equally improved and chances for life about even, so far as we could determine. The one who returned to the favorable climate continued to gain, whereas the one who remained here died within three months.

Another rule, which has proven true with both the cases mentioned and most of my other tubercular patients, is that the winter months seem even more favorable to them than the summer months. The fact is, I feel ready to take the position, and trust that others will sustain me, that we can just as safely send our patients, who are in the first or beginning the second stage of tuberculosis, to the North Woods in the dead of winter as in the balmy spring or warm summer months.

During the past two years, a goodly number of the tubercular patients under my care have been induced to take up their abode in the region drained by the Oswegatchie; although some have died, and perhaps others will eventually, I find that each has been materially improved, and those who have died only succumbed to the disease when, as in one of the cases mentioned, again brought under the influences of a bad climate.

It does not appear necessary that they should be in the heart of the woods or on the top of the mountains, but it does appear necessary that there should be a porous soil, so that they can breathe dry, pure air both day and night. I will admit that the tubercular patients that come to my notice are mostly laryngeal cases, but a great majority of them have such pulmonary complications that the situation is more or less serious. I will admit, also, that there may be other localities, in or near New York state, where these patients might do well.

As you well know I do not stand alone in being an advocate of the North Woods as a health resort. Other men of larger experience in tubercular therapeutics have written upon the same subject —notably Dr. Alfred L. Loomis, who, as early as 1879, wrote an article on the Adirondack region as a therapeutical agent in the treatment of pulmonary phthisis.

I simply want to place this paper before you for what little it is worth, hoping that it may help impress upon the profession the value of our Adirondack forests as a winter health resort, and the fact that in that region we have a natural, all-the-year-round sanitarium for consumptives.

The conclusions drawn from experience and the authorities consulted are:

*First*—That a thorough rubbing in of lactic acid, in the proper strength and at regular intervals, works nicely in allaying local tubercular ulcerations.

*Second*—That a continuous residence, or as near so as possible, with systematic bathing and exercise, in an altitude of from 1,000 to 2,000 feet, together with a porous soil and adjacent forests, will greatly benefit and perhaps cure even quite advanced tubercular manifestations.

*Third*—That we have here at home, in the Adirondacks of our own state, localities where many tubercular patients may find the climatic and physical environment required. In fact, my personal opinion is that they will receive more benefit there than from a residence in Florida, and full as much as could be obtained in Colorado or Southern California.

*Fourth*—That the patients do equally as well in fall and winter and gain more in weight than in the summer months; hence, we need not keep them here for "spring to open," but should insist upon an immediate removal, where practicable, from unfavorable climatic influences.

117 E. Jefferson Street.

